# Supplementary material for: Multi‐dataset identification of innovative feature genes and molecular mechanisms in keratoconus
Source: J Cell Mol Med. 2024 Sep 19;28(18):e70079. doi: 10.1111/jcmm.70079 (PMC11412914; doi:10.1111/jcmm.70079)
Supplement: Supplementary file 2 — Material S2. [file JCMM-28-e70079-s002.zip › 7.GSEA analysis/1.ARL11/KEGG_JAK_STAT_SIGNALING_PATHWAY.html]

Details for gene set KEGG\_JAK\_STAT\_SIGNALING\_PATHWAY[GSEA]

|  || Dataset | ARL11.group.cls#h\_versus\_l |
| Phenotype | group.cls#h\_versus\_l |
| Upregulated in class | l |
| GeneSet | KEGG\_JAK\_STAT\_SIGNALING\_PATHWAY |
| Enrichment Score (ES) | -0.29859692 |
| Normalized Enrichment Score (NES) | -0.99966294 |
| Nominal p-value | 0.48897797 |
| FDR q-value | 1.0 |
| FWER p-Value | 1.0 |
Table: GSEA Results Summary

  

Fig 1: Enrichment plot: KEGG\_JAK\_STAT\_SIGNALING\_PATHWAY      
 Profile of the Running ES Score & Positions of GeneSet Members on the Rank Ordered List

  

| SYMBOL | TITLE | RANK IN GENE LIST | RANK METRIC SCORE | RUNNING ES | CORE ENRICHMENT || 1 | IL7R | na | 136 | 0.619 | 0.0241 | No |
| 2 | IL21 | na | 309 | 0.534 | 0.0434 | No |
| 3 | CCND2 | na | 468 | 0.484 | 0.0608 | No |
| 4 | LEP | na | 970 | 0.392 | 0.0641 | No |
| 5 | IL3RA | na | 1221 | 0.364 | 0.0734 | No |
| 6 | IFNL3 | na | 1401 | 0.346 | 0.0839 | No |
| 7 | IL26 | na | 1410 | 0.345 | 0.0993 | No |
| 8 | JAK3 | na | 1845 | 0.308 | 0.1007 | No |
| 9 | PIK3R3 | na | 2180 | 0.284 | 0.1039 | No |
| 10 | IL2RB | na | 2535 | 0.263 | 0.1056 | No |
| 11 | PIK3CG | na | 2870 | 0.247 | 0.1072 | No |
| 12 | IL4R | na | 3015 | 0.240 | 0.1139 | No |
| 13 | IFNA6 | na | 3473 | 0.220 | 0.1106 | No |
| 14 | IL3 | na | 3483 | 0.220 | 0.1203 | No |
| 15 | OSM | na | 3598 | 0.215 | 0.1268 | No |
| 16 | IL7 | na | 3809 | 0.208 | 0.1301 | No |
| 17 | CBLB | na | 3964 | 0.202 | 0.1348 | No |
| 18 | IFNA14 | na | 4269 | 0.191 | 0.1347 | No |
| 19 | IL19 | na | 4416 | 0.186 | 0.1389 | No |
| 20 | SOCS3 | na | 4502 | 0.183 | 0.1447 | No |
| 21 | IFNA16 | na | 4914 | 0.169 | 0.1405 | No |
| 22 | STAT4 | na | 5954 | 0.138 | 0.1167 | No |
| 23 | PIK3R1 | na | 6079 | 0.135 | 0.1192 | No |
| 24 | SOS1 | na | 6194 | 0.132 | 0.1219 | No |
| 25 | IFNA4 | na | 6226 | 0.131 | 0.1270 | No |
| 26 | IL2RA | na | 6518 | 0.124 | 0.1242 | No |
| 27 | IL6 | na | 6613 | 0.121 | 0.1270 | No |
| 28 | SOCS7 | na | 7129 | 0.109 | 0.1170 | No |
| 29 | CSH1 | na | 7456 | 0.103 | 0.1122 | No |
| 30 | EPO | na | 7618 | 0.099 | 0.1121 | No |
| 31 | CREBBP | na | 7701 | 0.098 | 0.1141 | No |
| 32 | IFNE | na | 7843 | 0.096 | 0.1144 | No |
| 33 | GHR | na | 8677 | 0.080 | 0.0939 | No |
| 34 | IL23R | na | 8686 | 0.080 | 0.0973 | No |
| 35 | PIK3R2 | na | 8783 | 0.078 | 0.0981 | No |
| 36 | IL20RA | na | 9017 | 0.075 | 0.0947 | No |
| 37 | AKT2 | na | 9348 | 0.070 | 0.0883 | No |
| 38 | STAT5B | na | 9483 | 0.068 | 0.0875 | No |
| 39 | SOS2 | na | 9955 | 0.061 | 0.0767 | No |
| 40 | IL13 | na | 10076 | 0.059 | 0.0759 | No |
| 41 | PIK3R5 | na | 10118 | 0.059 | 0.0774 | No |
| 42 | IRF9 | na | 10270 | 0.057 | 0.0756 | No |
| 43 | MYC | na | 10444 | 0.054 | 0.0730 | No |
| 44 | CNTF | na | 10488 | 0.054 | 0.0742 | No |
| 45 | IL15RA | na | 10561 | 0.053 | 0.0746 | No |
| 46 | STAT6 | na | 11447 | 0.042 | 0.0509 | No |
| 47 | IL13RA1 | na | 11669 | 0.040 | 0.0463 | No |
| 48 | IL20RB | na | 11926 | 0.037 | 0.0405 | No |
| 49 | IFNL2 | na | 12511 | 0.031 | 0.0251 | No |
| 50 | TSLP | na | 12541 | 0.031 | 0.0256 | No |
| 51 | CBL | na | 12670 | 0.030 | 0.0233 | No |
| 52 | JAK2 | na | 13146 | 0.026 | 0.0107 | No |
| 53 | IL12A | na | 13401 | 0.024 | 0.0044 | No |
| 54 | PIK3CA | na | 13512 | 0.023 | 0.0023 | No |
| 55 | EPOR | na | 13761 | 0.021 | -0.0039 | No |
| 56 | SPRY1 | na | 13842 | 0.020 | -0.0053 | No |
| 57 | STAT5A | na | 14398 | 0.016 | -0.0207 | No |
| 58 | CTF1 | na | 14511 | 0.015 | -0.0232 | No |
| 59 | PTPN11 | na | 14607 | 0.014 | -0.0253 | No |
| 60 | IL15 | na | 14630 | 0.014 | -0.0253 | No |
| 61 | IFNA2 | na | 15087 | 0.011 | -0.0380 | No |
| 62 | STAT1 | na | 15344 | 0.009 | -0.0451 | No |
| 63 | IL11RA | na | 15527 | 0.007 | -0.0500 | No |
| 64 | PIAS1 | na | 15768 | 0.005 | -0.0567 | No |
| 65 | IL22 | na | 15849 | 0.005 | -0.0588 | No |
| 66 | IFNAR1 | na | 15880 | 0.005 | -0.0594 | No |
| 67 | STAM | na | 15947 | 0.004 | -0.0612 | No |
| 68 | CCND1 | na | 16546 | -0.000 | -0.0785 | No |
| 69 | IL5RA | na | 16947 | -0.003 | -0.0899 | No |
| 70 | GH2 | na | 17060 | -0.004 | -0.0930 | No |
| 71 | STAT2 | na | 17227 | -0.005 | -0.0976 | No |
| 72 | AKT1 | na | 18085 | -0.011 | -0.1218 | No |
| 73 | SOCS4 | na | 18164 | -0.011 | -0.1236 | No |
| 74 | STAM2 | na | 18209 | -0.012 | -0.1243 | No |
| 75 | PIAS3 | na | 18227 | -0.012 | -0.1243 | No |
| 76 | PRL | na | 18902 | -0.016 | -0.1430 | No |
| 77 | JAK1 | na | 19011 | -0.017 | -0.1454 | No |
| 78 | CBLC | na | 20116 | -0.024 | -0.1762 | No |
| 79 | IL2RG | na | 20265 | -0.026 | -0.1793 | No |
| 80 | IL6R | na | 20441 | -0.027 | -0.1831 | No |
| 81 | IFNB1 | na | 20512 | -0.028 | -0.1839 | No |
| 82 | SPRY2 | na | 20969 | -0.031 | -0.1957 | No |
| 83 | CNTFR | na | 21425 | -0.035 | -0.2073 | No |
| 84 | IFNGR1 | na | 21859 | -0.038 | -0.2181 | No |
| 85 | PIAS2 | na | 22018 | -0.040 | -0.2208 | No |
| 86 | CISH | na | 22732 | -0.047 | -0.2393 | No |
| 87 | GRB2 | na | 23043 | -0.051 | -0.2460 | No |
| 88 | PIAS4 | na | 23627 | -0.058 | -0.2602 | No |
| 89 | PTPN6 | na | 23706 | -0.058 | -0.2598 | No |
| 90 | TPO | na | 23798 | -0.060 | -0.2598 | No |
| 91 | PIK3CB | na | 23818 | -0.060 | -0.2576 | No |
| 92 | EP300 | na | 23862 | -0.060 | -0.2561 | No |
| 93 | SPRED1 | na | 24149 | -0.064 | -0.2615 | No |
| 94 | IL6ST | na | 24197 | -0.065 | -0.2599 | No |
| 95 | CCND3 | na | 24389 | -0.068 | -0.2623 | No |
| 96 | IL12RB1 | na | 24647 | -0.071 | -0.2665 | No |
| 97 | CSF3R | na | 24746 | -0.073 | -0.2661 | No |
| 98 | LIF | na | 25088 | -0.077 | -0.2724 | No |
| 99 | STAT3 | na | 25255 | -0.079 | -0.2736 | No |
| 100 | PIM1 | na | 25511 | -0.083 | -0.2772 | No |
| 101 | IL9R | na | 25644 | -0.085 | -0.2772 | No |
| 102 | PIK3CD | na | 25759 | -0.087 | -0.2766 | No |
| 103 | IL10RA | na | 26348 | -0.096 | -0.2892 | No |
| 104 | IFNAR2 | na | 26387 | -0.096 | -0.2860 | No |
| 105 | LEPR | na | 26569 | -0.100 | -0.2867 | No |
| 106 | SOCS1 | na | 26619 | -0.101 | -0.2835 | No |
| 107 | IL12B | na | 26869 | -0.106 | -0.2859 | No |
| 108 | SOCS5 | na | 26944 | -0.107 | -0.2832 | No |
| 109 | IL23A | na | 27477 | -0.118 | -0.2932 | Yes |
| 110 | IFNGR2 | na | 27521 | -0.119 | -0.2891 | Yes |
| 111 | AKT3 | na | 27547 | -0.120 | -0.2844 | Yes |
| 112 | IL10RB | na | 27648 | -0.122 | -0.2817 | Yes |
| 113 | IL12RB2 | na | 27665 | -0.123 | -0.2766 | Yes |
| 114 | IL21R | na | 27687 | -0.123 | -0.2716 | Yes |
| 115 | TYK2 | na | 28528 | -0.144 | -0.2894 | Yes |
| 116 | CSF2RB | na | 28575 | -0.145 | -0.2841 | Yes |
| 117 | IL9 | na | 28748 | -0.151 | -0.2823 | Yes |
| 118 | MPL | na | 28953 | -0.155 | -0.2811 | Yes |
| 119 | IL22RA1 | na | 28959 | -0.155 | -0.2742 | Yes |
| 120 | BCL2L1 | na | 29160 | -0.161 | -0.2727 | Yes |
| 121 | SPRED2 | na | 29334 | -0.167 | -0.2702 | Yes |
| 122 | CLCF1 | na | 29418 | -0.170 | -0.2649 | Yes |
| 123 | LIFR | na | 29466 | -0.171 | -0.2585 | Yes |
| 124 | GH1 | na | 29711 | -0.179 | -0.2574 | Yes |
| 125 | CSF3 | na | 29731 | -0.180 | -0.2498 | Yes |
| 126 | CSF2 | na | 30130 | -0.191 | -0.2526 | Yes |
| 127 | SPRY4 | na | 30257 | -0.196 | -0.2474 | Yes |
| 128 | IL2 | na | 30766 | -0.213 | -0.2524 | Yes |
| 129 | CRLF2 | na | 30784 | -0.214 | -0.2432 | Yes |
| 130 | IFNA8 | na | 31555 | -0.242 | -0.2545 | Yes |
| 131 | IFNW1 | na | 31920 | -0.257 | -0.2534 | Yes |
| 132 | SPRY3 | na | 32017 | -0.261 | -0.2444 | Yes |
| 133 | OSMR | na | 32318 | -0.275 | -0.2406 | Yes |
| 134 | IFNLR1 | na | 32364 | -0.279 | -0.2292 | Yes |
| 135 | IL5 | na | 32563 | -0.292 | -0.2217 | Yes |
| 136 | IFNL1 | na | 32599 | -0.294 | -0.2094 | Yes |
| 137 | CSF2RA | na | 32761 | -0.305 | -0.2002 | Yes |
| 138 | IL10 | na | 32834 | -0.310 | -0.1882 | Yes |
| 139 | IFNA10 | na | 33230 | -0.339 | -0.1843 | Yes |
| 140 | IFNA7 | na | 33254 | -0.341 | -0.1695 | Yes |
| 141 | IL24 | na | 33469 | -0.357 | -0.1595 | Yes |
| 142 | PRLR | na | 33554 | -0.365 | -0.1454 | Yes |
| 143 | IFNK | na | 33679 | -0.376 | -0.1319 | Yes |
| 144 | IL4 | na | 33709 | -0.380 | -0.1155 | Yes |
| 145 | SOCS2 | na | 33805 | -0.390 | -0.1006 | Yes |
| 146 | IFNG | na | 33859 | -0.398 | -0.0841 | Yes |
| 147 | IL22RA2 | na | 34109 | -0.438 | -0.0714 | Yes |
| 148 | IFNA21 | na | 34187 | -0.451 | -0.0532 | Yes |
| 149 | IL11 | na | 34287 | -0.471 | -0.0347 | Yes |
| 150 | IL13RA2 | na | 34307 | -0.475 | -0.0137 | Yes |
| 151 | IL20 | na | 34569 | -0.570 | 0.0046 | Yes |
Table: GSEA details [plain text format]

  

Fig 2: KEGG\_JAK\_STAT\_SIGNALING\_PATHWAY      
 Blue-Pink O' Gram in the Space of the Analyzed GeneSet

  

Fig 3: KEGG\_JAK\_STAT\_SIGNALING\_PATHWAY: Random ES distribution      
 Gene set null distribution of ES for **KEGG\_JAK\_STAT\_SIGNALING\_PATHWAY**
